# The positive effects of positive coping on mental health in college students during the COVID-19 campus lockdown

**DOI:** 10.3389/fpubh.2023.1267347

**Published:** 2023-11-22

**Authors:** Meiqi Li, Lijun Chen

**Affiliations:** School of Psychology, Shandong Normal University, Jinan, Shandong, China

**Keywords:** COVID-19, campus lockdown, coping style, mental health, crisis

## Abstract

**Objectives:**

Isolation has been an effective method to control the spread of COVID-19 over the past 3 years. However, lifestyle changes may have a negative impact on mental health. To examine the effects of positive coping on mental health in college students during the COVID-19 campus lockdown, this study conducted an online cross-sectional survey.

**Methods:**

In October 2022, following a prolonged campus lockdown of nearly 3 years, 313 university students from a university in Shandong Province, China, were invited to complete an online questionnaire. The questionnaire comprised a self-administered general situation questionnaire, the Simple Coping Style Scale, and the Hospital Depression and Anxiety Scale.

**Results:**

(1) The analysis of variance revealed a significant main effect of coping Style on depression [F(4,300) = 2.446, *p* = 0.047] during the COVID-19 campus lockdown. A *post-hoc* test indicated that college students who engaged in study (*p* = 0.012) or sports (*p* = 0.027) during their free time had significantly lower depression scores than those who used the Internet. (2) Independent sample t-tests showed significant differences in positive and negative coping styles among college students in terms of depression (*t* = 6.891, *p* < 0.001) and anxiety scores (*t* = 7.745, *p* < 0.001). (3) Pearson correlation analysis demonstrated a negative correlation between positive coping style and anxiety (*r* = −0.378, *p* < 0.001), and between positive coping style and depression (*r* = 0.427, *p* < 0.001). Positive correlations were also found between the negative coping style and anxiety (*r* = 0.155, *p* = 0.007), and between the negative coping style and depression (*r* = 0.190, *p* < 0.001).

**Discussion:**

The study suggested that fostering positive coping in students can mitigate mental health issues during crises, providing a blueprint for university mental health initiatives during epidemics.

## Introduction

The COVID-19, as a public health emergency, has had extensive and lasting effects on people's mental health, especially for the college students who were locked up on campus for a long time. After the outbreak of COVID-19, some studies have found that the proportion of depression and anxiety among college students was very high (1, 2). Some studies have even found that 51 and 45.9% of students have anxiety and depression, respectively (3). Fortunately, studies have found positive mental health variables in alleviating symptoms of psychological distress such as depression and anxiety among college students during COVID-19. Biber et al. (4) explored data from college students during COVID-19 and found that more optimism and gratitude are closely related to lower anxiety (4). Research by Michelle Haikalis et al. suggests that subjects who perceive greater positive changes brought about by COVID-19 have alleviated increases in anxiety and depression (5). Research by Seyedmohammad Mirhosseini et al. found that life satisfaction is a protective factor in preventing depression during the pandemic (6). It suggested that positive coping strategies such as engaging in more positive activities or taking a more positive perspective on situations might reduce the damage to the mental health.

Coping has been identified as a crucial factor influencing individuals' mental health amidst the COVID-19 pandemic (7, 8). It is characterized by a relatively stable yet dynamic style or feature that adapts to various circumstances. In critical situations, effective coping strategies can serve as protective factors against mental health issues. It's noteworthy that individuals employing specific coping styles exhibit varying mental health outcomes. Coping styles can be broadly classified into positive and negative (9, 10). Research indicates that negative coping styles are linked with mental health problems, whereas method-oriented coping strategies are associated with improved mental health compared to avoidance coping strategies (11–14). College students may respond to the pandemic through activities such as exercise, internet usage, or studying. Exercise has been positively correlated with positive coping styles. Regular exercisers tend to employ positive coping styles more frequently, potentially reducing anxiety levels (15). Conversely, adolescents spending extensive time online have been found to be more prone to depression (16). However, dedicating more leisure time to studying can alleviate academic stress, subsequently reducing depressive symptoms (17–20). Studies have demonstrated that positive problem responses in community samples are associated with lower depression levels, while negative responses correlate with higher depression levels (21, 22).

As a result of COVID-19, people's mental health is significantly affected (23), and individual coping styles can buffer the effects of the external environment on mental health (24). College students, as the most mobile and active group of students, are not yet fully mature in ideology and psychology. Consequently, they are severely affected. However, during the COVID-19 pandemic, a significant crisis event, it remains uncertain whether the individual coping styles of college students confined to campus have a significant positive or negative impact on depression and anxiety levels. Answering this question will enable us to implement appropriate measures to assist college students in maintaining their mental health during such incidents. Therefore, to understand whether coping styles and what coping strategies having an impact on depression and anxiety status of college students during the COVID-19 campus closure, this study recruited 313 college students who were incarcerated in schools and explored the relationship between coping and depression and anxiety during the period of epidemic interdiction.

We proposed three hypotheses. First, as the duration of COVID-19 increases, the depression and anxiety levels of college students should decrease compared to the initial few months of COVID-19. Second, we hypothesized that during COVID-19, college students who adopted positive coping styles such as studying or exercising would exhibit lower depression and anxiety scores. In contrast, those who adopted negative coping styles such as internet surfing would exhibit higher depression or anxiety scores. Third, we also hypothesized a significant association between college students' coping styles and depression and anxiety levels. We expected a negative correlation between positive coping style and depression or anxiety level, and a positive correlation between negative coping styles and depression or anxiety levels.

## Materials and methods

The data of this project were collected from a university in China (Shandong Normal University). After a long time campus lockdown (nearly 3 years), a total of 313 questionnaires were distributed. After excluding a few participants who filled out the questionnaire incorrectly due to carelessness, finally 305 valid questionnaires were collected, resulting in an effective rate of 97.44%. The participants' ages ranged from 18 to 26 years, with an average age of 20.05 ± 0.91 years. The sample included 116 males and 189 females. Data was collected via an online platform named “wjx” by the end of October 2022. Online informed consent was obtained from all participants prior to completing the online questionnaires. The study was approved by the local ethical committee of School of Psychology, Shandong Normal University and all research activities were adhered to the principles of the Declaration of Helsinki.

### Measures

#### General questionnaire

On the “wjx” platform, we developed a general questionnaire covering gender, age, exercise, study, Internet access, and other ways of coping, as well as the impact of COVID-19 on certain inconveniences.

#### Simple coping style scale

A simplified coping style questionnaire (SCSQ) was used to investigate individual coping style tendencies. This scale was developed based on a comprehensive understanding of coping styles at home and abroad in conjunction with the characteristics of China's population. It has good reliability and validity among Chinese college students, with a Cronbach's alpha coefficient of 0.9 (10). There were 20 items in total, including two dimensions of positive and negative coping. The dimension of positive coping was composed of items 1–12, which mainly reflected the characteristics of positive coping. The dimension of negative coping was composed of items 13–20, which mainly reflected the characteristics of negative coping. In the questionnaire, a four-level scale ranging from 0 to 3 was used. The higher the positive coping score, the more inclined the respondents are to adopt positive coping styles. The higher the negative coping score, the more likely the respondents were to adopt negative coping styles. The Cronbach's α for this scale in this study was 0.852.

#### Hospital depression and anxiety scale

In order to assess individual depression and anxiety, the Hospital Anxiety and Depression Scale (HADS) was utilized. According to Zigmond and Snaith (25), the scale was universal and consisted of 14 items, seven of which were related to depression, and seven to anxiety. In the Chinese population, the scale also has good reliability and validity 24 with a Cronbach'sα coefficient of 0.879 (26). There were four options available for each item, marked by “0 “, “1 “, “2 “, and “3 “. The anxiety and depression items crossed, and their total scores were calculated by overlaying the two sets of items. A higher score indicates that the subjects are suffering from depression or anxiety to a greater extent. The Cronbach's α for this scale in this study was 0.854.

### Statistical analysis

Data analysis was conducted using SPSS version 22.0. The independent samples *t*-test was used to compare means between two groups, while one-way ANOVA was employed to compare means among multiple groups. Pearson correlation analysis was utilized for correlation analysis. All statistical tests in this study were conducted at a significance level of *p* < 0.05.

## Results

### A general profile of study participants

The survey revealed that the majority of students (*n* = 191 or 62.6%) spent their free time surfing the Internet during the COVID-19 campus lockdown. This was followed by studying (47 students, 15.4%), chatting with classmates (31 students, 10.2%), exercising (28 students, 9.2%), and other ways (8 students, 2.6%). According to the survey, 135 students (44.3%) believed that COVID-19 had a significant impact on going home from school and going out to play, while 103 students (33.8%) thought it had a very significant impact. For details, see Table 1.

**Table 1:** General information (*N* = 305).

		**Number of people**	**Composition ratio (%)**
How to spend free time during the COVID-19	Study	47	15.4
	Internet surfing	191	62.6
	Exercise	28	9.2
	Chatting with classmates	31	10.2
	Others	8	2.6
How much of an effect on going home from school and going out to play	No reduction in frequency	4	1.3
	No impact	28	9.2
	Basically no impact	35	11.5
	Had some impact	135	44.3
	Had a significant impact	103	33.8

### The results measured by the HADS

The results indicated that 21% of college students (*n* = 64) scored 8 or more on the anxiety scale, while 15.4% of college students (*n* = 47) scored 8 or more on the depression scale. This suggests that 15.4% of college students may be experiencing depression and 21.0% may be dealing with anxiety. The detection rates of depression and anxiety in this study are lower than they were in the initial few months of the pandemic (30.7 and 23.9%, respectively) (2), which supports the first hypothesis.

### Depression and anxiety scores with different coping styles during the COVID-19 campus lockdown

**Figure 1:**
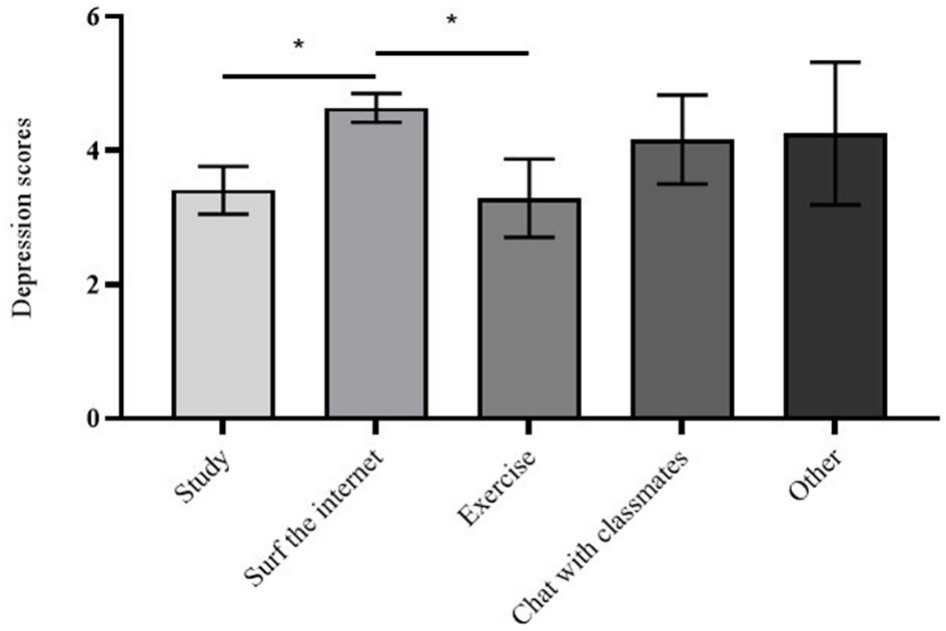
Differences in Depression Scores among Different Coping Ways during the COVID-19 campus lockdown. *p < 0.05. Error bars represent SE.

In this study, studying and exercising are considered positive coping strategies, while surfing the Internet and chatting are considered negative strategies. A one-way ANOVA was used to evaluate the differences in depression and anxiety scores among college students who spent their free time in different ways during the COVID-19 campus lockdown. There was a significant difference in depression scores [F(4,300) = 2.446, *p* = 0.047] and a non-significant difference in anxiety scores [F(4,300) = 0.817, *p* = 0.515] among college students who spent their free time in different ways during the lockdown. Pairwise comparisons showed significant differences in depression scores between studying and surfing the Internet (*p* = 0.012), with students who spent their free time studying having significantly lower depression scores than those who spent their free time surfing the Internet. There were also significant differences between surfing the Internet and exercising (*p* = 0.027), with students who spent their free time surfing the Internet having significantly higher depression scores than those who spent their free time exercising. For details, see Table 2 and Figure 1.

**Table 2:** ANOVA analysis of different coping styles on depression and anxiety scores.

	**Study (*n =* 47)**	**Surf the NET (*n =* 191)**	**Do sports (*n =* 28)**	**Chat with classmate (*n =* 31)**	**Other (*n =* 8)**	***F* (4,300)**	***p***
Depression	3.40 ± 2.45	4.63 ± 2.98	3.29 ± 3.09	4.16 ± 3.71	4.25 ± 3.01	2.446^*^	0.047
Anxiety	4.57 ± 3.20	5.19 ± 3.27	4.46 ± 3.35	4.65 ± 3.20	6.00 ± 4.78	0.817	0.515

^*^p < 0.05.

**Figure 2:**
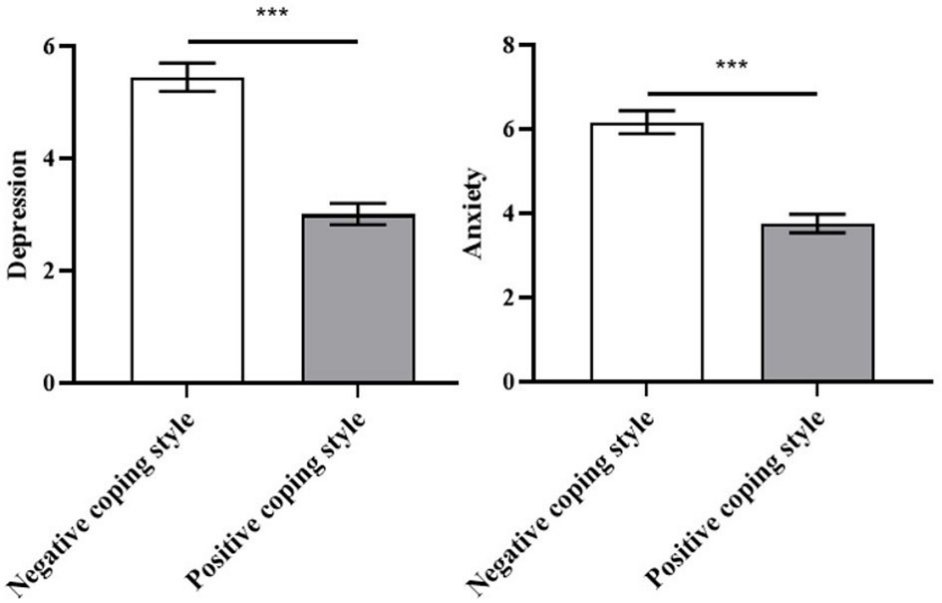
Comparison of differences in depression and anxiety scores between two coping style tendencies. ***p < 0.001. Error bars represent SE.

To compare the depression and anxiety scores of students who tend to use positive coping with those who tend to use negative coping, independent sample t-tests were performed. It was found that students who used negative coping styles scored significantly higher in depression and anxiety than students who used positive coping styles (*t* = 7.745, *p* < 0.001; *t* = 6.891, *p* < 0.001; see Table 3 and Figure 2). These findings support the second hypothesis.

**Table 3:** *T*-tests for two coping style tendencies on depression and anxiety scores.

	**Positive coping style (*n =* 148)**	**Negative coping style (*n =* 157)**	***t***	***p***
Depression	3.01 ± 2.29	5.45 ± 3.16	7.745^***^	0.000
Anxiety	3.76 ± 2.65	6.17 ± 3.43	6.891^***^	0.000

^***^p < 0.001.

### Correlation analysis

The Pearson correlation was used to analyze the correlation between coping styles and mental health status. The results revealed a negative correlation between positive coping style and anxiety (*r* = −0.378, *p* < 0.001), as well as between positive coping style and depression (*r* = −0.427, *p* < 0.001). Positive correlations were also found between negative coping style and anxiety (*r* = 0.155, *p* = 0.007), and between negative coping style and depression (*r* = 0.190, *p* < 0.001). Please refer to Table 4 for more details. These findings support the third hypothesis.

**Table 4:** Correlation between depression, anxiety and coping style.

	**Anxiety**	**Depression**	**Positive cope**	**Negative cope**
Anxiety	1			
Depression	0.663^***^	1		
Positive cope	−0.378^***^	−0.427^***^	1	
Negative cope	0.155^**^	0.190^***^	0.208^***^	1

^**^p < 0.01; ^***^p < 0.001.

## Discussion

During the campus lockdown period, this study utilized the Simple Coping Style Scale (SCSQ) and the Hospital Depression and Anxiety Scale (HADS) to conduct an online survey assessing the impact of coping styles on the mental health of college students. The result showed that the detection rate of anxiety and depression among college students were 21.0 and 15.4%, respectively. These rates were lower than those at the first few months of the pandemic (30.7 and 23.9%) (2). This decrease suggests that state-imposed controls and blockades on the COVID-19 pandemic were effective, and that mental health may gradually recover during later stages of a public health disaster (27). Moreover, as COVID-19 has mutated several times and risks have gradually reduced, people have adapted to the epidemic environment. This adaptation, along with initial infectious disease control measures, may improve young people's mental health (28). In addition, various psychological protective factors such as positive coping and psychological resilience might contribute to the gradual recovery of mental health (4–6).

This study found that students with negative coping styles had higher depression and anxiety scores than those with positive coping styles. Positive coping styles were significantly negatively correlated with depression and anxiety levels, while negative coping styles were significantly positively correlated with these levels. This is consistent with previous studies (29, 30), indicating that positive coping styles continue to have a protective effect on college students' mental states during the COVID-19 campus lockdown. This study found that students with negative coping styles had higher depression and anxiety scores than those with positive coping styles. There was a significant correlation between coping style scores and depression and anxiety scores. Positive coping styles were significantly negatively correlated with depression and anxiety levels, while negative coping styles were significantly positively correlated with these levels. This is consistent with previous studies, indicating that positive coping styles continue to have a protective effect on college students' mental states during the COVID-19 campus lockdown (31). According to the theory of social cognitive stress, adopting a positive coping style may reduce an individual's negative emotions, while adopting a negative coping style may exacerbate an individual's negative emotions and reduce their subjective well-being (32, 33). Our results support this theory. Therefore, methods such as cognitive-behavioral interventions aimed at improving an individual's positive coping ability may be crucial for maintaining mental health in various stressful events. This study also investigated whether specific coping methods impact mental health during the pandemic. We examined the relationship between certain positive and negative coping methods and mental health. The results indicated that students who spent their free time on the Internet (often viewed negatively) during campus lockdown had significantly higher depression scores than those who spent their free time studying and exercising (often viewed positively). A particular syndrome, known as “headline stress disorder,” is characterized by anxiety due to continuous news media coverage (34). During campus lockdowns, students could not leave school, making the Internet the most accessible means of connecting with the outside world. Consequently, they were more likely to encounter numerous epidemic reports online, including rumors. Some students struggled to discern the truth of this information, leading to increased anxiety and depression (35). Academic engagement describes the level of commitment students have toward their learning activities, which significantly influences their academic performance (36–38). Self-efficacy refers to an individual's belief in their ability to accomplish specific tasks (39). Academic self-efficacy is the belief in one's capability to handle various learning tasks (40). There is a strong positive correlation between academic self-efficacy and academic engagement, which may be mutually reinforcing (41). When students spend their free time studying, it is likely that their academic engagement increases and their academic self-efficacy improves. Higher self-efficacy is associated with lower depression levels; thus, students with higher academic self-efficacy tend to have lower depression levels (42). Studies have shown that exercise interventions positively impact psychological disorders such as anxiety and depression in college students (43–45). Therefore, students who choose positive coping methods like studying or exercising during their free time might experience lower levels of depressive mood. This finding underscored the importance of effective management of students' leisure time during campus closures. Schools could organize or guide students to engage in positive extracurricular activities to maintain a healthier psychological state.

In conclusion, our research suggested that enhancing positive coping styles among college students may reduce their susceptibility to psychological issues during crises. Specifically, our study offered coping strategies for university students: by dedicating more of their free time to learning and physical activities, students could mitigate stress during such unique crisis events. However, this study has two limitations. Firstly, all participants were undergraduates from a Normal University, which may not provide a sufficiently random and representative sample. Secondly, this study was cross-sectional. The survey was conducted at the end of October 2022, during the ongoing development of the COVID-19 pandemic. This dynamic process means that the coping styles, depression, and anxiety of college students at different stages of the pandemic's progression were not comprehensively explored.

## Data availability statement

The raw data supporting the conclusions of this article will be made available by the authors, without undue reservation.

## Ethics statement

The studies involving humans were approved by the Local Ethical Committee of School of Psychology, Shandong Normal University. The studies were conducted in accordance with the local legislation and institutional requirements. The participants provided their written informed consent to participate in this study.

## Author contributions

ML: Data curation, Visualization, Writing – original draft. LC: Resources, Supervision, Writing – review & editing.
